# Redox-Related Proteins in Melanoma Progression

**DOI:** 10.3390/antiox11030438

**Published:** 2022-02-22

**Authors:** Larissa A. C. Carvalho, Rodrigo G. Queijo, Alexandre L. B. Baccaro, Ádamo D. D. Siena, Wilson A. Silva, Tiago Rodrigues, Silvya Stuchi Maria-Engler

**Affiliations:** 1 Department of Clinical and Toxicological Analysis, School of Pharmaceutical Sciences, University of São Paulo, Avenida Professor Lineu Prestes, 580, São Paulo 05508-00, SP, Brazil; lari.anastacio@gmail.com (L.A.C.C.); rodrigogoncalvesqueijo@usp.br (R.G.Q.); 2 Centro de Pós-Graduação e Pesquisa Oswaldo Cruz, Faculdade Oswaldo Cruz, Rua Brigadeiro Galvão, 535, Sao Paulo 01151-000, SP, Brazil; alexandre.baccaro@gmail.com; 3 Department of Genetics, Ribeirão Preto Medical School, University of São Paulo, Avenida Bandeirantes, 3900, Ribeirao Preto 14049-900, SP, Brazil; adamo@usp.br (Á.D.D.S.); wilsonjr@usp.br (W.A.S.J.); 4 Center for Natural and Human Sciences, Federal University of ABC, Avenida dos Estados, 5001, Santo Andre 09210-580, SP, Brazil; trodrigues.ufabc@gmail.com

**Keywords:** melanoma, resistance, redox proteins, oxidative stress, antioxidants

## Abstract

Melanoma is the most aggressive type of skin cancer. Despite the available therapies, the minimum residual disease is still refractory. Reactive oxygen and nitrogen species (ROS and RNS) play a dual role in melanoma, where redox imbalance is involved from initiation to metastasis and resistance. Redox proteins modulate the disease by controlling ROS/RNS levels in immune response, proliferation, invasion, and relapse. Chemotherapeutics such as BRAF and MEK inhibitors promote oxidative stress, but high ROS/RNS amounts with a robust antioxidant system allow cells to be adaptive and cooperate to non-toxic levels. These proteins could act as biomarkers and possible targets. By understanding the complex mechanisms involved in adaptation and searching for new targets to make cells more susceptible to treatment, the disease might be overcome. Therefore, exploring the role of redox-sensitive proteins and the modulation of redox homeostasis may provide clues to new therapies. This study analyzes information obtained from a public cohort of melanoma patients about the expression of redox-generating and detoxifying proteins in melanoma during the disease stages, genetic alterations, and overall patient survival status. According to our analysis, 66% of the isoforms presented differential expression on melanoma progression: NOS2, SOD1, NOX4, PRX3, PXDN and GPX1 are increased during melanoma progression, while CAT, GPX3, TXNIP, and PRX2 are decreased. Besides, the stage of the disease could influence the result as well. The levels of PRX1, PRX5 and PRX6 can be increased or decreased depending on the stage. We showed that all analyzed isoforms presented some genetic alteration on the gene, most of them (78%) for increased mRNA expression. Interestingly, 34% of all melanoma patients showed genetic alterations on TRX1, most for decreased mRNA expression. Additionally, 15% of the isoforms showed a significant reduction in overall patient survival status for an altered group (PRX3, PRX5, TR2, and GR) and the unaltered group (NOX4). Although no such specific antioxidant therapy is approved for melanoma yet, inhibitors or mimetics of these redox-sensitive proteins have achieved very promising results. We foresee that forthcoming investigations on the modulation of these proteins will bring significant advances for cancer therapy.

## 1. Introduction

Melanoma is one of the most aggressive tumors currently known [1]. More than 100,000 new cases afflicting both sexes were estimated in the US during 2020. It is the fifth most prevalent cancer in men and in women, corresponding to 7 and 5 % of all estimated new cases of cancer. Its incidence increases, although it has begun to decline in recent birth cohorts [2]. Besides, a decline in yearly mortality has also been observed and might be ascribed to the higher survival of patients due to the new immunotherapy treatment with ipilimumab, the first immune checkpoint inhibitor approved for cancer therapy, and the targeted therapy with vemurafenib, a BRAF inhibitor (BRAFi) for the treatment of advanced melanoma approved by the Food and Drug Administration (FDA) in 2011. Still, the development of drug resistance is a significant clinical challenge to be overcome. The decline in mortality rate highlights the importance of investing in new cancer control interventions and in research to promote better treatment options [2]. Recent studies have suggested ROS/RNS increased production, oxidative stress, and the redox imbalance as the significant causes of melanomagenesis and melanoma resistance [3,4,5,6]. Thus, understanding these redox processes in melanoma development and resistance could help achieve better approaches for improved therapy.

The first event of melanoma is the formation of a benign nevus, which is the most pronounced proliferation of healthy melanocytes. This nevus is a uniformly colored lesion, with a regular pigmentation pattern in a circle and a tan or darkish-brown background. The nevus can become dysplastic, which is the anomalous development and is usually asymmetrical, with irregular edges containing various colors or increased diameters. A dysplastic nevus can evolve to radial growth, and cells proliferate intra-epidermically. Dysplastic nevus presenting elevated lesions already show intraepidermal cancer, and the cells can penetrate the dermis. In vertical growth, cells have the ability to invade the dermis, and form an expansive nodule, extending into the reticular dermis and fat. The last event is metastasis, in which cells successfully spread to other skin areas and other organs, proliferating, and establishing a metastatic focus [7].

In melanoma, ROS are generated as a consequence of (i) increased metabolism of transformed cells, (ii) immune response against the tumor, (iii) UV irradiation, (iv) melanin production, and (v) an altered antioxidant system [8,9]. Vemurafenib itself promoted mitochondrial fusion in melanoma cells [10] and increased oxidative metabolism and ROS production [11]. The FDA also approved the use of combined BRAF and MEK inhibitors, which have doubled the time of progression, but also leads to relapse. In fact, resistance to BRAF and MEK inhibitors is related to the oxidative metabolism [12,13,14]. It has been suggested to induce oxidative stress to make resistant cells more vulnerable [15]. Still, disruption of redox balance enhances the effects of BRAF-inhibition in melanoma [16]. In this sense, overcoming targeted therapy resistance is a major concern in melanoma field.

The higher amounts of ROS/RNS generated by cancer cells subsidize the molecular changes that drive tumor initiation, promotion, progression, and chemoresistance [17]. Reactive species act as signaling mechanisms for proliferation, vascular function, and wound healing at physiological levels. Lower levels enable cell cycle arrest, while higher levels can drive cells to death [17].

Tumor-promoting functions of ROS/RNS comprise (i) tumorigenesis: according to the concentration and duration of exposure, ROS can drive genomic instability, act downstream to the activation of oncogenes or inactivation of tumor suppressor genes, modulate activities of signaling pathways involved in tumor proliferation (PI3K and MAPK) and they also may induce epigenetic alterations by methylating and inactivating tumor suppressor genes; (ii) angiogenesis: ROS can affect angiogenesis by mediating endothelial cell proliferation, migration, and tube formation, and they also modulate VEGF signaling; (iii) invasion and metastasis: subtle increases in steady-state H_2_O_2_ levels can lead to pro-migratory signaling in cancer, endogenous growth factors, and cytokines which are known to display critical functions in the invasiveness (some mediating ROS generation ROS). ROS also participate in cell migration by modulating the actin cytoskeleton. It can oxidize/inhibit phosphotyrosine phosphatase (PTP) and prevent the enzyme from dephosphorylating and inactivating FAK (involved in controlling cell motility). Additionally, it is involved in the redox-dependent degradation of extracellular matrix by several mechanisms; (iv) chemoresistance: ROS could modulate critical proteins involved in therapy resistance [17]. In addition, redox switches can lead the transformation status of quiescent to proliferative cells [18]. ROS/RNS may contribute to aggressiveness, self-renew, antiproteases inhibition, local tissue injuring and tumor heterogeneity promotion [19,20]. Consequently, more aggressive cancer cells, such as melanoma cells, have higher levels of ROS than normal healthy cells [5,19].

However, ROS/RNS can also act as a tumor-suppressor agent. Although higher amounts of ROS/RNS could be beneficial to tumorigenesis and resistance, the augmented antioxidant defense in these cells indicates that decreasing antioxidant defense and increasing ROS/RNS levels further to a toxic level may push cells beyond the breaking point, suppressing tumor development and providing a unique opportunity to eliminate the tumor. This could be accomplished by using drugs that increase ROS production, inhibit the antioxidant defense, or a combination of both [17]. In this sense, ROS/RNS can activate several cell-death pathways, so several agents were reported to effectively eradicate and sensitize cancer cells to chemotherapeutic agents via modulating ROS/RNS production. It is well accepted that ROS/RNS can eliminate tumor cells via apoptosis, autophagy, necroptosis, and ferroptosis (a novel type of non-apoptotic, non-autophagic, and non-necroptotic form of cell death) [17,19,21,22].

In this line, decreasing ROS/RNS levels or using antioxidants could also be considered a promising alternative to prevent ROS-mediated tumor development. Enzymatic and non-enzymatic components form the antioxidant defense system. Non-enzymatic antioxidants comprise carotenoids, ascorbic acid, vitamin D derivatives, flavonoids, N-acetyl cysteine (NAC), α-tocopherol, and other small molecules. Conversely, enzymatic antioxidants are redox-sensitive proteins, like superoxide dismutase (SOD), peroxiredoxin (PRX), thioredoxin system (TRX), glutathione peroxidase (GPX), glutathione reductase (GR), and catalase (CAT) [11,19,20,23].

In view of the aspects mentioned above, the exact role of ROS/RNS in cancer is still contradictory and context-dependent. Once ROS/RNS and antioxidants can promote or suppress tumors, new therapies that induce or inhibit such species may present advantages and disadvantages [17]. A better understanding of the mechanisms involved in these treatments may allow the development of new promising strategies. Besides, understanding the redox balance involved in cancer is still a challenge.

## 2. Materials and Methods

In order to analyze the expression of genes in melanoma progression, public microarray data initially published by Scatolini et al. (2010) [24] was used. In this data set, there are samples from 18 common nevi, 11 dysplastic nevi, 8 from a radial phase of melanoma growth, 15 from vertical phase of melanoma growth and 5 from melanoma metastases. These data were downloaded from the Gene Expression Omnibus–GEO repository (https://www.ncbi.nlm.nih.gov/geo/ accessed on 10 September 2019), under the code GSE12391. The statistical analysis corresponds to the Analysis of Variance (ANOVA) followed by the Tukey post-hoc test. All analyzed data and the images generated were obtained using the R platform and Prism GraphPad 7 software. For the patient’s data analysis, we chose to keep the sample classifications as already mentioned by the reference paper and investigate gene expression at different stages of the disease, i.e., benign nevus, dysplastic nevus, radial growth, vertical growth, and metastatic melanoma. Such classification is interesting since it provides insights into the role of the genes during the disease progression, and they can be used as biomarkers. To analyze the genetic alterations and overall patient survival status in cutaneous skin melanoma, the cBioPortal for Cancer Genomics was used. The cBioPortal for Cancer genomics is an open-access resource: http://www.cbioportal.org/ accessed on 28 June 2021 [25,26]. The name of the genes was searched in the cBioPortal database for skin cutaneous melanoma (TCGA, Firehose Legacy), *n* = 479 samples. The selected genomic profiles were mRNA expression and protein expression with a z-score threshold of 1.5.

## 3. Results

### 3.1. RNS and ROS-Generating Enzymes

#### 3.1.1. Nitric Oxide Synthase (NOS)

Nitric oxide (NO) is a free radical that participates in melanoma signaling. Elevated NO levels correlate with poor outcomes, but intermediate levels can limit tumor cell proliferation. Therefore, the synthesis of NO by nitric oxide synthase (NOS) plays an essential role in melanoma progression [27]. NOS catalyzes the reaction of L-arginine and oxygen with NADPH as co-substrate to produce L-citrulline and NO. Humans express three isoforms of NOS, namely neuronal NOS (nNOS or NOS1), inducible NOS (iNOS or NOS2), and endothelial NOS (eNOS or NOS3). The eNOS and nNOS isoforms are grouped as constitutively active NOS (cNOS), while iNOS is induced by immunologic stimuli [28,29]. Normal melanocytes express both cNOS and iNOS [30].

Current literature proposes that NO displays a controversial role in melanoma by inducing or inhibiting apoptosis, with both pro- and anti-tumorigenic activities (reviewed in 27). It was shown that NOS activity and levels are significantly higher in melanoma than in melanocytes [31,32,33]. Previous studies indicated that iNOS promoted tumor proliferation [34,35,36], and it was associated with poor patient survival and increased resistance to cisplatin [37]. It was found that nNOS was expressed in 49% of benign nevi, 72% of atypical nevi, and 82% of primary malignant cells, suggesting its role in melanoma progression [38]. Thus, nNOS is increased in melanoma and is associated with a more proliferative profile [39,40], possibly contributing to cell escape from apoptosis [40]. The eNOS could be involved in melanoma development, since its uncoupling is an important source of superoxide that drives the malignant transformation [41].

Specific nNOS and iNOS inhibitors exhibited promising efficacy against both in vitro and pre-clinical melanoma mouse models [39,42,43,44], and reduced proliferation of melanoma cells [39,43,44,45]. The use of extracellular NO donor or intracellular expression of iNOS revealed that NO exposure supports growth/survival in melanoma [46]. On the other hand, inducing iNOS with evodiamine increases NO production and drives apoptosis [47]. Once there are still some contradictory data about the pro- or anti-tumorigenic effects of NO inhibition, probably due to the several levels that it can achieve, further studies should be accomplished to clarify its real role in melanoma.

**Figure 1:**
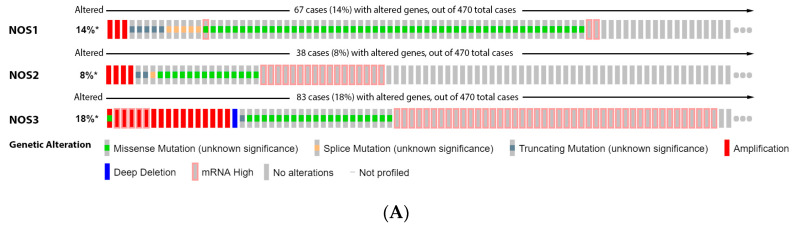

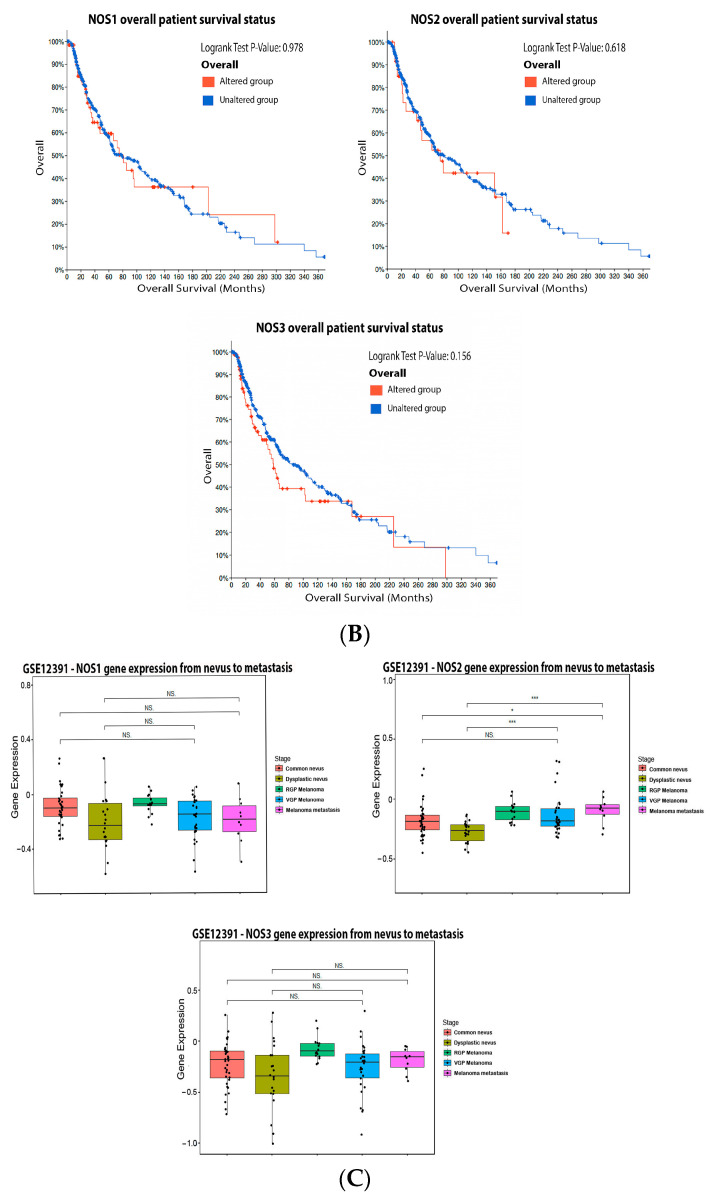
Nitric oxide synthase on melanoma. (**A**) Percentage of patients with altered genes and types of genetic alterations; (**B**) overall patient survival status, and (**C**) expression in melanoma progression. Red boxes represent the common nevus, yellow boxes the dysplastic nevus, green boxes the radial growth phase (RGP) melanoma, blue boxes the vertical growth phase (VGP) melanoma, and purple boxes the metastatic melanoma. The statistical analysis was performed by ANOVA followed by Tukey test, with *** *p* < 0.001, * *p* < 0.05 when compared to common or dysplastic nevus. NS: Not significant.

Furthermore, our data analysis showed that 14% of the melanoma patients have genetic alteration on NOS1 gene, 8% on NOS2 and 18% on NOS3. Higher mRNA was the most prevalent alteration of NOS2 and NOS3, while missense mutation was more prevalent for NOS1 (Figure 1A). However, none of these isoforms caused significative change in patient survival (Figure 1B). Our data from patient biopsies, showed that there were no significant alterations in the NOS1 and NOS3 expression levels during melanoma progression (Figure 1C). However, the expression of NOS2 was significantly increased in the vertical growth phase when compared to dysplastic nevus and in metastatic melanoma once compared to the common and dysplastic nevus (Figure 1C).

#### 3.1.2. NADPH Oxidase (NOX)

The NADPH oxidase (NOX) family comprises a class of flavoenzymes that uses one or two electrons of NADPH to reduce oxygen to superoxide radical (O_2_^•−^) and H_2_O_2_, across biological membranes. There are seven NOX isoforms in mammals, i.e., NOX1–NOX5, and the dual oxidases DUOX-1 and DUOX-2 [48]. NOX4, DUOX-1 and DUOX-2 produce H_2_O_2_, while NOX1-3 and NOX5 produce O_2_^•−^ [49]. Among the NOX family, NOX1 and NOX4 isoforms play a major role in melanoma, while NOX5 is also important but still scarcely studied [50].

The expression and activity of NOX1 are increased in melanoma cells and their stages [19,51]. Its overexpression increases melanoma invasiveness, while the knockdown or inhibition decreases it. Indeed, NOX1 seems to be involved in epithelial–mesenchymal transition (EMT) [51]. NOX4 expression is increased in several melanoma cell lines. It is associated with metastasis, and its silencing decreases cell growth and tumorigenicity in nude mice. The use of DPI and antioxidants blocked melanoma cell proliferation, and the knockdown of NOX4 induced G2-M cell cycle arrest [52]. Pharmacological NOX inhibition and its silencing decreased the viability of the human melanoma cell line, and induced cellular shape changes by disassembling focal adhesion processes through the FAK pathway [53]. NOX5 is overexpressed in melanoma [50]. Its overexpression in UACC-257 melanoma cells increases cell growth, BrdU positive cells, γ-H2AX levels, normoxic HIF-1α expression, and decreased p27Kip1 expression. In contrast, the knockdown of NOX5 decreases cell growth, HIF-1α expression, Akt and GSK3β phosphorylation, and increases p27Kip1 expression. Therefore, the expression of NOX5 supports cell proliferation by generating ROS that modulates HIF-1α and AKT/GSk3β/p27Kip1 [54]. Once it was revealed that NOX enzymes played a critical role in melanoma, they were used as a targeted therapy [19,55,56]. There are several NOX inhibitors that hold great potential against melanoma. The inhibition of NOX with DPI decreased ROS production, inhibited cell growth, promoted cell differentiation of B16 melanoma cells, and increased MITF expression [57].

**Figure 2:**
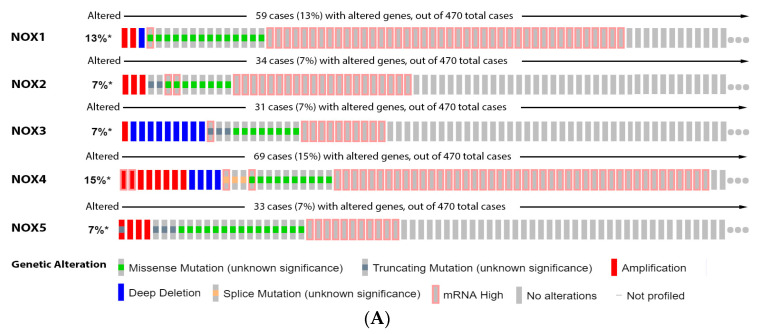

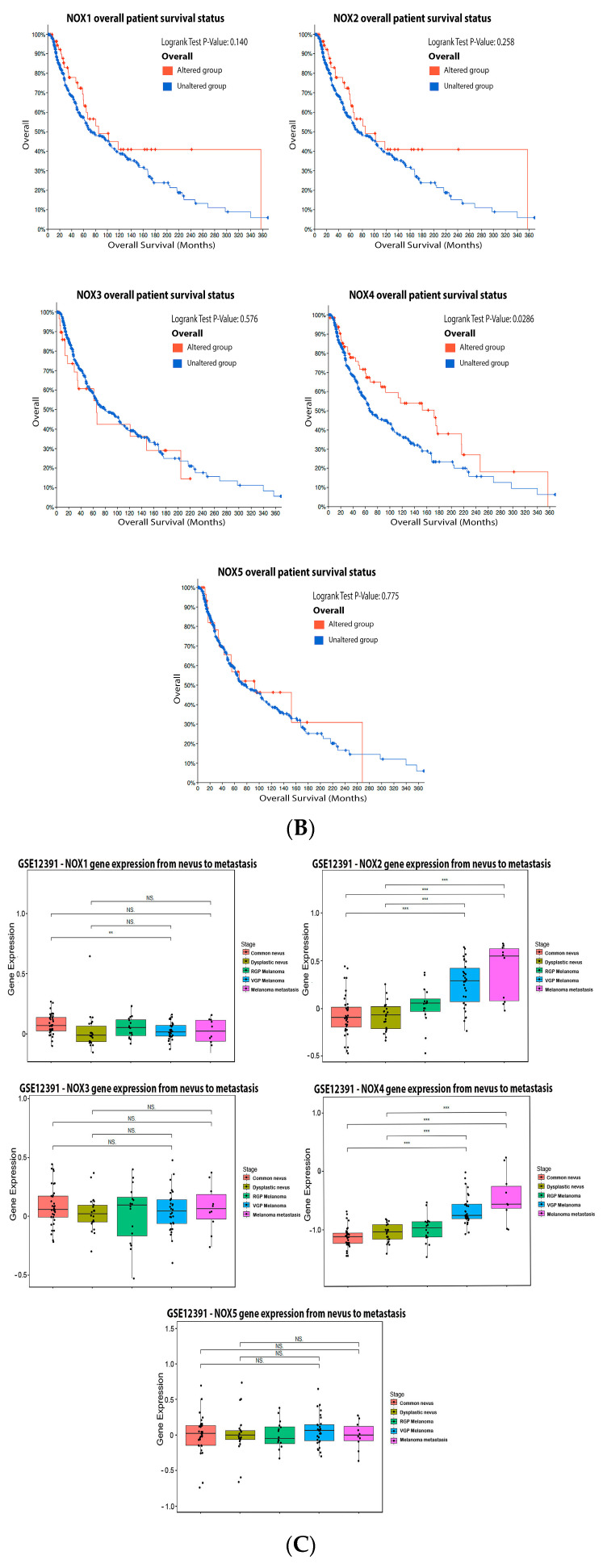
NADPH oxidases on melanoma. (**A**) Percentage of patients with altered genes and types of genetic alterations; (**B**) overall patient survival status, and (**C**) expression in melanoma progression. Red boxes represent the common nevus, yellow boxes the dysplastic nevus, green boxes the radial growth phase (RGP) melanoma, blue boxes the vertical growth phase (VGP) melanoma, and purple boxes the metastatic melanoma. The statistical analysis was performed by ANOVA followed by Tukey test, *** *p* < 0.001, ** *p* < 0.01, * *p* < 0.05 when compared to common or dysplastic nevus. NS: Not significant.

In this regard, we showed that 13% of the melanoma patients have alterations on NOX1 gene, 7% on NOX2, NOX3 and NOX5, and 15% on NOX4. The most prevalent genetic alterations were increased mRNA expression for NOX1–4 and missense mutation for NOX5 (Figure 2A). Only the altered group of patients with mutations on NOX4 demonstrated significative increased survival (Figure 2B). Our data analysis points out to the lower NOX1 expression in VGP melanoma than in common nevus (Figure 2C), while no significant differences were found for NOX3 and NOX5 at different stages (Figure 2C). Moreover, NOX2 and NOX4 expression was increased during melanoma progression (Figure 2C).

### 3.2. RNS and ROS-Detoxifying and Sensitive Enzymes

#### 3.2.1. Superoxide Dismutase (SOD)

Superoxide dismutases are enzymes that catalyze the dismutation reaction to convert O_2_^•−^ into H_2_O_2_. There are three described SOD isoforms, i.e., SOD1 (CuZnSOD) located in the cytoplasm, SOD2 (MnSOD) in the mitochondria, and SOD3 (ecSOD) in the extracellular compartment [58].

Several studies proposed that SOD2 activity or expression is altered in skin cancers, although its role is still controversial in melanoma. It was shown that lower levels of SOD2 is related to metastasis [59]. Besides, an increased expression of SOD2 suppressed the malignant phenotype of melanoma cells in vitro, and also led to the loss of the colony-forming ability [60]. In contrast, Schadendorf showed that serum SOD2 level was elevated in melanoma patients and, since it was correlated to the disease progression, they proposed SOD2 as a tumor marker and a sensitive biomarker in serum for monitoring melanoma [61]. The alterations in SOD expression are involved in therapy resistance as well [62]. Though few studies are addressing the role of SOD1 and SOD3 in melanoma, it was shown that SOD1 is involved in melanogenesis and/or differentiation [63,64]. The overexpression of SOD3 inhibited the growth of B16F1 melanoma cells [65]. Considering that ROS are involved in cellular proliferation signaling, antioxidant therapies using SOD mimetics are currently being explored [66]. However, it is also important to consider that depending on the ROS levels; it might help the tumor progression.

**Figure 3:**
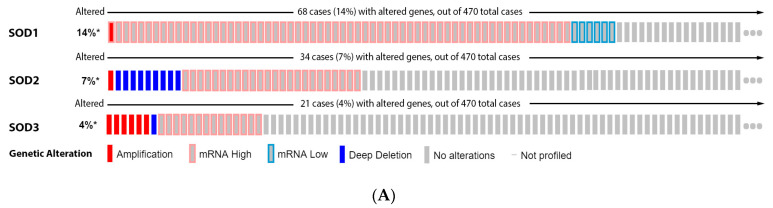

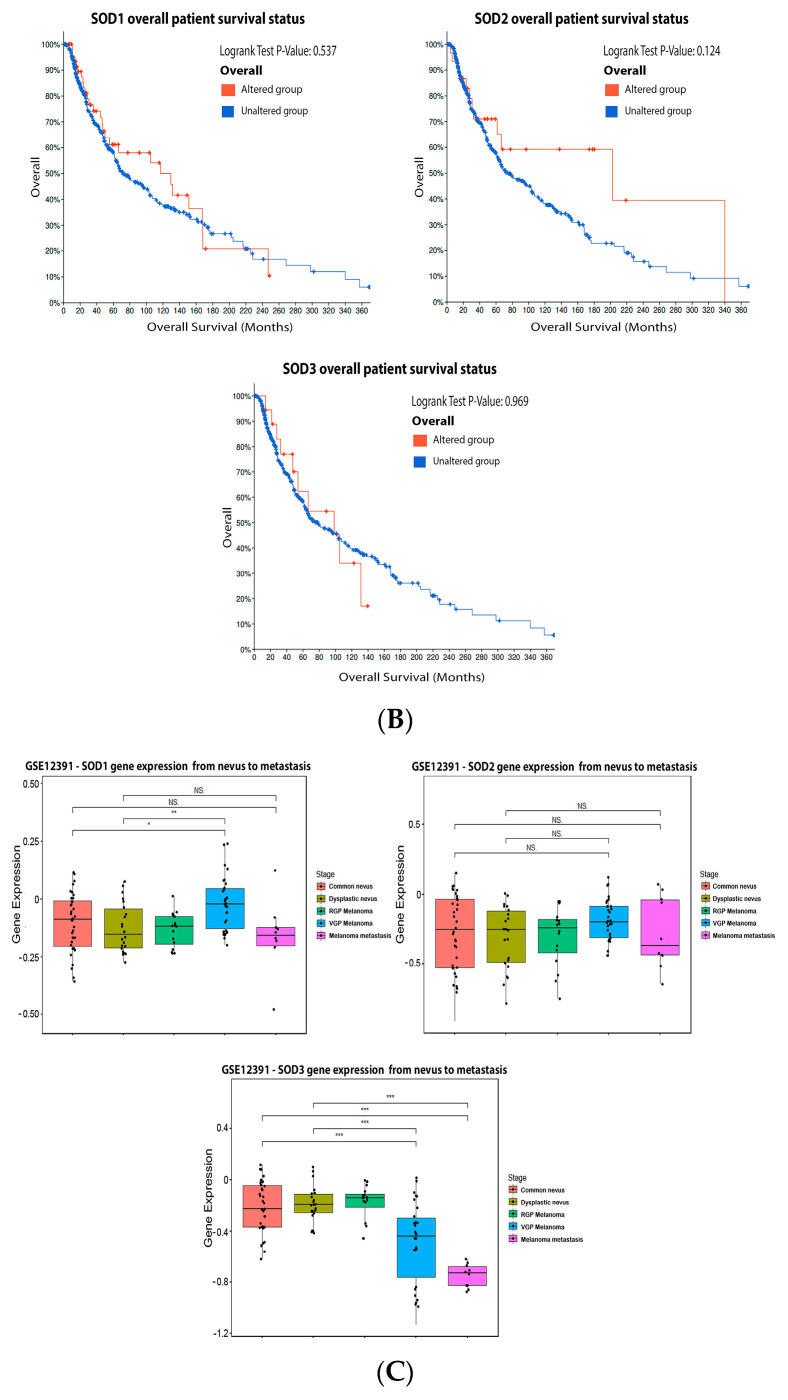
Superoxide dismutase on melanoma. (**A**) Percentage of patients with altered genes and types of genetic alterations; (**B**) overall patient survival status, and (**C**) expression in melanoma progression. Red boxes are common nevus, yellow boxes are dysplastic nevus, green boxes are radial growth phase (RGP) melanoma, blue boxes are vertical growth phase (VGP) melanoma, and purple boxes are metastatic melanoma. The statistical analysis was performed by ANOVA followed by Tukey test, *** *p* < 0.001, ** *p* < 0.01, * *p* < 0.05 when compared to common or dysplastic nevus. NS: Not significant.

From our data analysis, 14% of the melanoma patients showed alteration on the SOD1 gene, 7% on SOD2, and 4% on SOD3. The most prevalent mutation was increased mRNA expression (Figure 3A). None of the isoforms had a significant impact on patient survival (Figure 3B). SOD1 expression increases from a common nevus and dysplastic nevus to melanoma vertical growth phase (Figure 3C), while SOD2 has no significant difference (Figure 3C). Moreover, we found that SOD3 was downregulated in melanoma when compared to nevus (Figure 3C).

#### 3.2.2. Catalase (CAT)

Catalase is a peroxidase-type enzyme that converts two H_2_O_2_ to O_2_ and H_2_O. Healthy melanocytes from melanoma patients exhibited decreased catalase activity and increased SOD activity, suggesting a redox imbalance during the melanoma progression [67]. Compared to CHO cells, melanoma cells presented decreased activities of catalase and SOD [68]. Moreover, another study proposed that catalase activity was increased in stages I, II, and III, but not in stage IV [69], evidencing a protective role of catalase in metastasis. A microarray in melanoma cells with different ROS levels after overexpression of catalase showed that dissimilar phenotypes were generated by differential compensation to hydrogen peroxide scavenging. These gene signatures were shown to promote melanogenesis or resemble less aggressive tumor cells by improving the antioxidant system, demonstrating that redox imbalance could control non-aggressive and metastatic melanomas [70].

Once catalase was shown to play a crucial role in melanoma, some studies using the cell-permeant polyethylene glycol conjugated catalase (PEG-catalase) have been published. It was shown that PEG-catalase inhibited cell survival, adhesion, invasion, and proliferation due to the induction of tumor dormancy [71]. The use of PEG-catalase prevented ROS-induced metastasis after surgical removal [71]. When melanoma cells were injected in mice and the animals were treated with PEG-catalase intravenously, almost a complete suppression of the growth of metastatic tumor in the lung was observed [72]. In this sense, despite some redox imbalance controversy of catalase, the use of PEG-catalase seems a promising therapy for melanoma.

**Figure 4:**
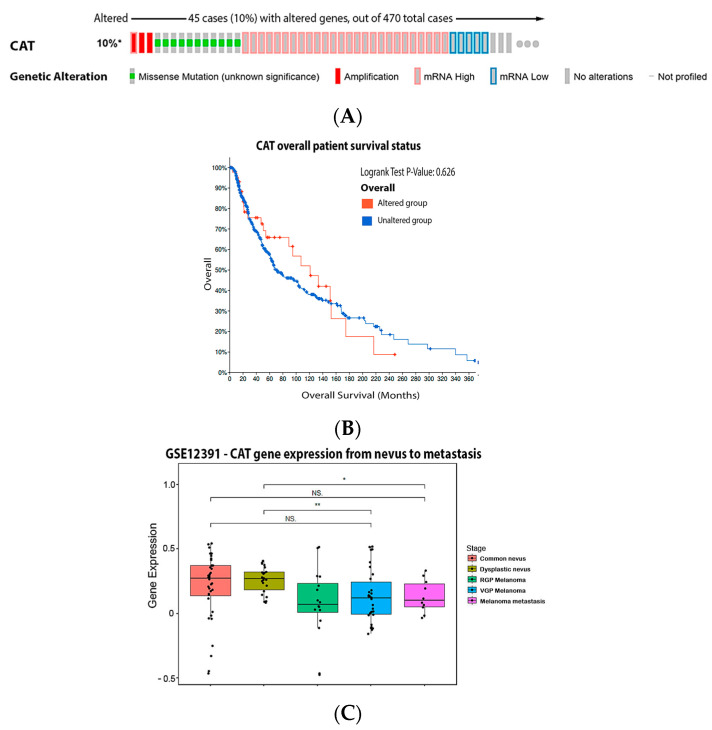
Catalase on melanoma. (**A**) Percentage of patients with altered genes and types of genetic alterations; (**B**) overall patient survival status, and (**C**) expression in melanoma progression. Red boxes represent the common nevus, yellow boxes the dysplastic nevus, green boxes the radial growth phase (RGP) melanoma, blue boxes the vertical growth phase (VGP) melanoma, and purple boxes the metastatic melanoma. The statistical analysis was performed by ANOVA followed by Tukey test, ** *p* < 0.01, * *p* < 0.05 when compared to common or dysplastic nevus. NS: Not significant.

Our analysis demonstrated that 10% of the melanoma patients have the mutation on the CAT gene and the most common was increased mRNA expression (Figure 4A). These mutations had no significant impact on survival (Figure 4B). Compared to dysplastic nevus, our data (Figure 4C) shows that catalase expression decreased through the vertical growth phase and metastatic melanoma.

#### 3.2.3. Glutathione System

Glutathione (γ-L-glutamyl-L-cysteinyl-glycine) is a tripeptide that can assume two redox conditions: reduced form (GSH) or oxidized disulfide form (GSSG). It is a key antioxidant against ROS, RNS, hypochlorous acid (HOCl), hydroxyl radicals (HO^•^), and other reactive oxidative species. It can act altogether with glutathione peroxidase (GPX), glutathione S-transferase (GST) and glutaredoxin (GLRX). GPX is responsible for the detoxification of organic and inorganic peroxides. Its activity depends on GSH, which is oxidized to GSSG. GST catalyzes the conjugation of GSH to a variety of endogenous and exogenous electrophilic compounds. They are divided into eight classes based on sequence homology: alpha (GSTA), theta (GSTT), mu (GSTM), omega (GSTO), kappa (GSTK), zeta (GSTZ), sigma (GSTS), and pi (GSTP). Glutaredoxins are small cytosolic enzymes, which reverse glutathione adducts on protein thiols. In all cases, the GSSG can be reduced by another enzyme called glutathione reductase (GR) using electrons from NADPH [73].

GSH is important to support redox homeostasis during melanin biosynthesis at the melanocytes [73]. A high GSH/GSSG ratio has an important role in metastatic progression [74,75,76]. Normal skin seems to have low redox capacity, whilst drug-sensitive melanoma cells have moderate redox capacity, and drug-resistant has high redox [16]. It has been demonstrated that melanoma cells are resistant to oxidative stress by maintaining elevated GSH, SOD, CAT and GPX levels [77,78]. Metastatic cells might consume GSH to keep redox homeostasis, once the GSH/GSSG ratio is lower in metastatic melanoma cells. Besides, it was shown that antioxidant therapy could promote invasion [76,79]. GSH promotes cell growth and resistance to therapy. Therefore, GSH depletion decreased the number and volume of invasive cells, and sensitizes melanoma to combination therapy [80]. It is well accepted that cells with decreased sensitivity to BRAFi present higher levels of antioxidant metabolites NADPH and GSH [16]. In fact, resistant cells adapt to oxidative stress by increasing GSH levels [13] and, in this context, drugs that oxidize or reduce GSH levels are under evaluation in clinical trials, in order to improve targeted therapy efficacy [81]. L-Buthionine-S-sulfoximine (BSO) is a selective and irreversible inhibitor of the enzyme γ-glutamylcysteine synthetase (γ-GCS) involved in the first step of GSH synthesis. Its use is associated with GSH depletion, and increased oxidative stress [82,83]. Additionally, BSO can downregulate GST expression, inhibit cell proliferation [72,84] and could also help to overcome melanoma resistance [85]. The depletion of GSH induced oxidative stress and apoptosis in melanoma cells [86].

GPX1 expression is augmented in melanoma. It is related to increased proliferation, and its depletion leads to a reduction of cellular growth [87]. GPX3 is considered an antioxidant protein and a possible tumor suppressor gene. It is downregulated in several tumors, including melanoma. Decreased levels of GPX3 are related to increased proliferation, motility, invasiveness, and poor prognosis [88]. Overexpression of GPX3 decreased the viability of melanoma cells and inhibited their growth in a xenografts model [89]. This data points to the important role of GPX3 inhibiting melanoma progression, and this subject should be more addressed in the field. Compared to the primary tumor, GST levels are decreased in metastasis derived from skin or lymph nodes. However, GST was increased with tumor progression, and GST π was the most expressed [90]. Its activity is correlated to malignity [91], and it is directly involved in melanoma invasion [92]. GSTs are related to the development of drug resistance in melanoma [93]. On the other hand, the isozymes GSTT and GSTM are absent in a large proportion of the population. They may affect the development of melanoma by decreasing oxidative stress [94]. GLRX is involved in angiogenesis and resistance [73,85,95,96,97,98]. The increased tumor progression in diabetes mellitus may be associated with GLRX upregulation [99]. GR is less studied, although it also seems to be relevant. The inhibition of GR generates oxidative stress and suppresses lung metastasis and subcutaneous growth of melanoma in vivo, and also decreases proliferation, colony formation, cell adhesion, migration, invasion in melanoma cells in vitro, blocks epithelial-to-mesenchymal transition (EMT) and affects actin rearrangement. In this sense, GR could act as a regulator of oxidative stress, and a potential target for melanoma therapy [100].

**Figure 5:**
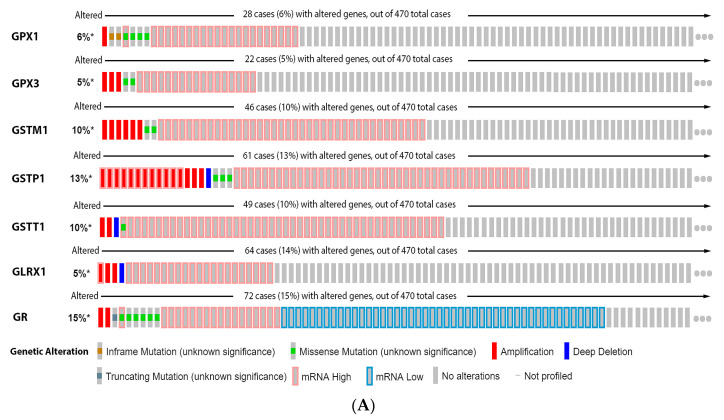

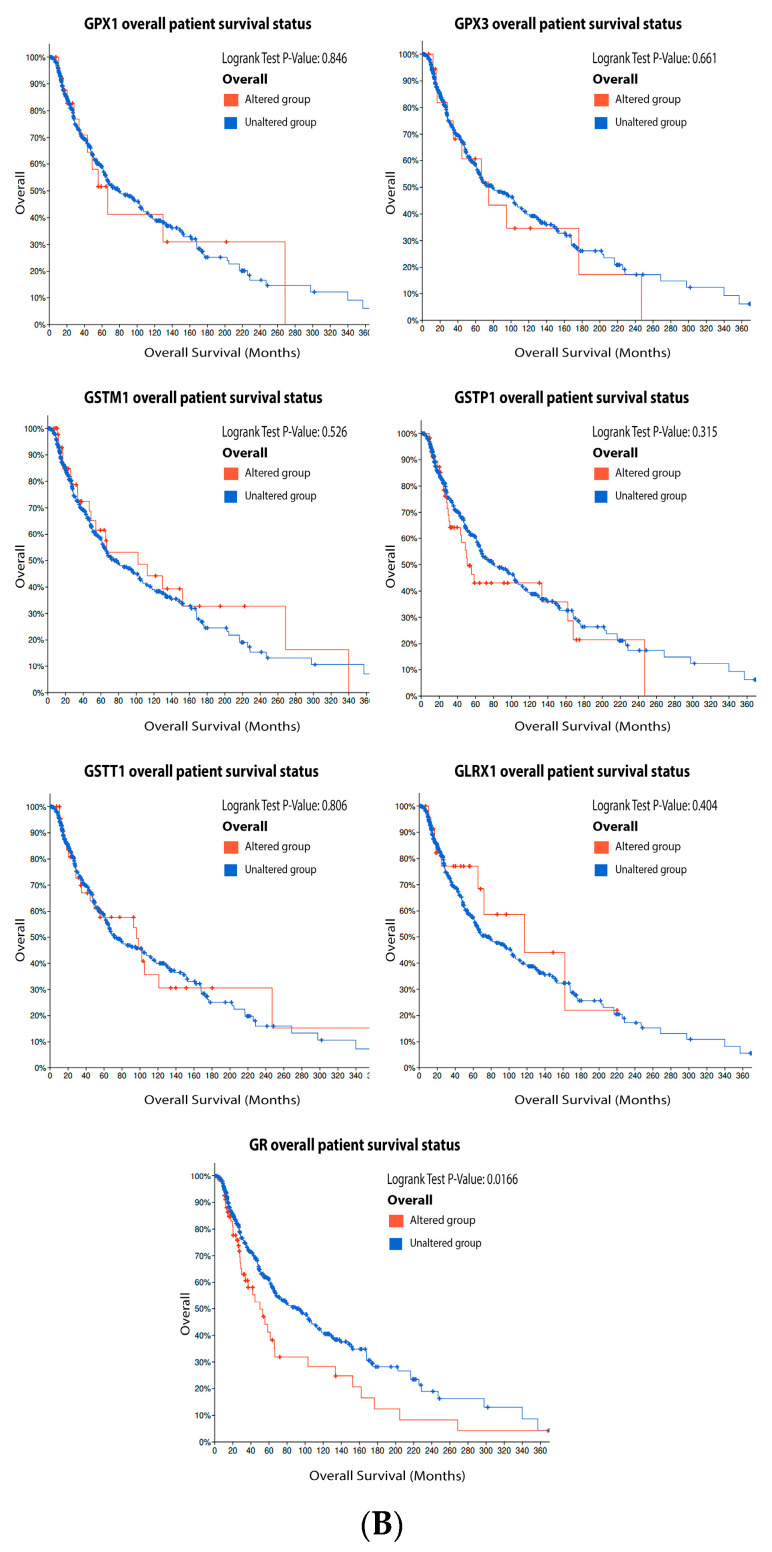

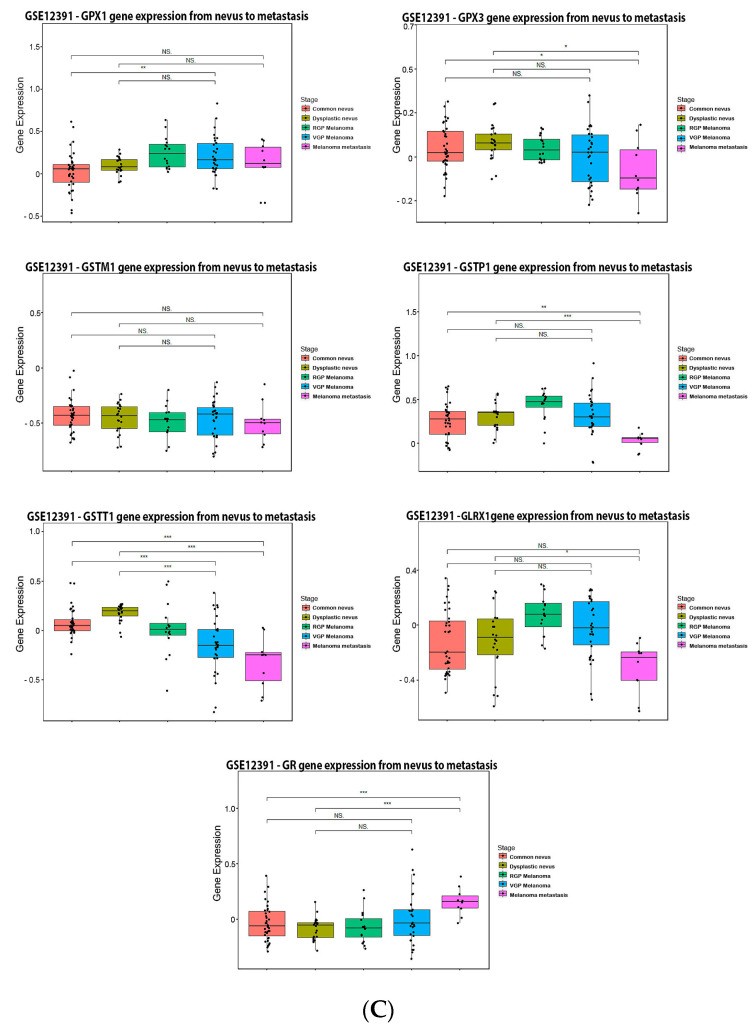
Glutathione-related enzymes on melanoma. (**A**) Percentage of patients with altered genes and types of genetic alterations; (**B**) overall patient survival status, and (**C**) expression in melanoma progression. Red boxes represent the common nevus, yellow boxes the dysplastic nevus, green boxes the radial growth phase (RGP) melanoma, blue boxes the vertical growth phase (VGP) melanoma, and purple boxes the metastatic melanoma. The statistical analysis was performed by ANOVA followed by Tukey test, *** *p* < 0.001, ** *p* < 0.01, * *p* < 0.05, when compared to common or dysplastic nevus. NS: Not significant.

It is possible to observe in our data analysis, presented in the Figure 5, that 6% of the patients have alteration on the GPX1 gene, 5% on GPX3, 10% on GSTM1, 13% on GSTP1, 10% on GSTT1, 5% on GLRX1, and 15% on GR. The most prevalent alteration was increased mRNA expression, except for GR that showed decreased mRNA expression (Figure 5A). Only GR alteration on patients had a significative decrease in survival (Figure 5B). Glutathione-related enzymes are differentially expressed in melanoma progression. GPX1 is increased in VGP melanoma compared to common nevus (Figure 5C). GPX3 is decreased in metastasis (Figure 5C). GSTM1 is not altered (Figure 5C). GSTP1 is decreased in metastasis compared to common and dysplastic nevus (Figure 5C). GSTT1 is decreased in VGP and metastatic melanoma compared to common and dysplastic nevus (Figure 5C). GLRX is decreased in metastatic melanoma compared to dysplastic nevus (Figure 5C). GR is increased in metastasis compared to common and dysplastic nevus (Figure 5C).

#### 3.2.4. Thioredoxin System

The thioredoxin system is a thiol-reducing system that comprises the thioredoxin (TRX) protein, thioredoxin reductase (TR), TXNIP (thioredoxin interacting protein), and NADPH (coenzyme α-nicotinamide adenine dinucleotide phosphate). TRX is a 12 kDa protein with conserved redox catalytic site (-Cys-Gly-Pro-Cys-), responsible for the maintenance of a reducing the intracellular environment state. Mammalian cells have two TRX isoforms: TRX1 present in cytosol and TRX2 in the mitochondria. TRX2 has two cysteines in its active site, while TRX1 has five cysteines and is important for NO signaling [101]. The reduced form of TRX is responsible for the reduction of several oxidized substrates. Besides its antioxidant property, it can translocate to the nucleus upon stimulation and associate with several transcription factors to regulate redox-sensitive genes. TR is a selenoenzyme that catalyzes the electron transfer to the oxidized form of TRX and other substrates, using reducing equivalents from NADPH [102]. TR has three isoforms in humans: TR1 present in the cytoplasm, TR2 or TGR in the testis, and TR3 in the mitochondria [101]. TXNIP is an endogenous inhibitor of TRX that binds to its redox-active site, negatively regulating its activity [101].

TRX is overexpressed in both nucleus and cytoplasm compared to tumors of a less aggressive nature [103] and seems to be involved in immune mechanisms in melanoma [104,105]. Overexpression of TXNIP increased transendothelial migration (TEM). Overexpression of TRX blocked hydrogen peroxide-induced TEM, demonstrating a cooperative role of TXNIP in melanoma progression that can be reversed by Trx [106]. Still, higher expression of TXNIP could play an important role in preventing metastasis [107,108]. TXNIP is downregulated after BRAFi treatment in melanoma, and overexpression of TXNIP in BRAFi resistant cells decreased migration and invasion [109]. TR1 levels correlate with melanoma progression, and targeting TR1 and glycolysis simultaneously, suppresses the growth of melanoma cells [110]. TR1 knockdown and pharmacological inhibition of TRX lead to decreased melanin levels and reduced activity of tyrosinase, involved in melanin synthesis. TR1 knockdown also caused a delay in upregulation of the melanocyte transcriptional factor MITF [111]. TR is overexpressed in many cancer types and has been recognized as an anti-cancer target. Several studies described the anticancer potential of TR inhibitors [112,113].

**Figure 6:**
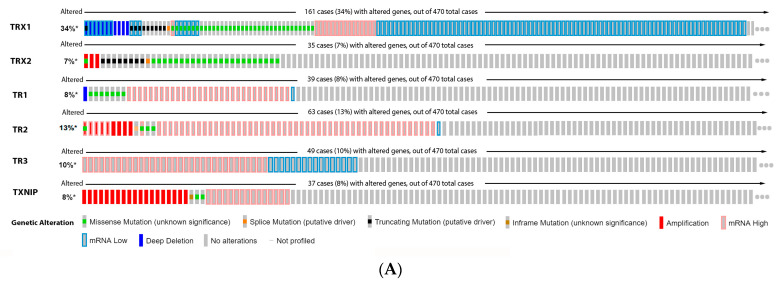

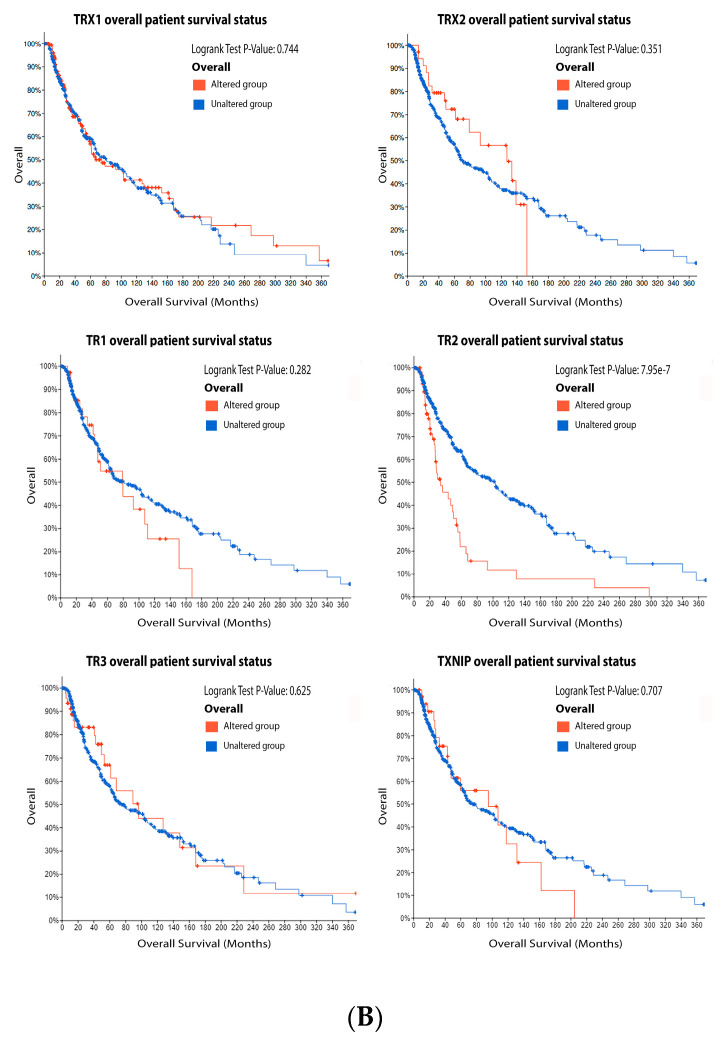

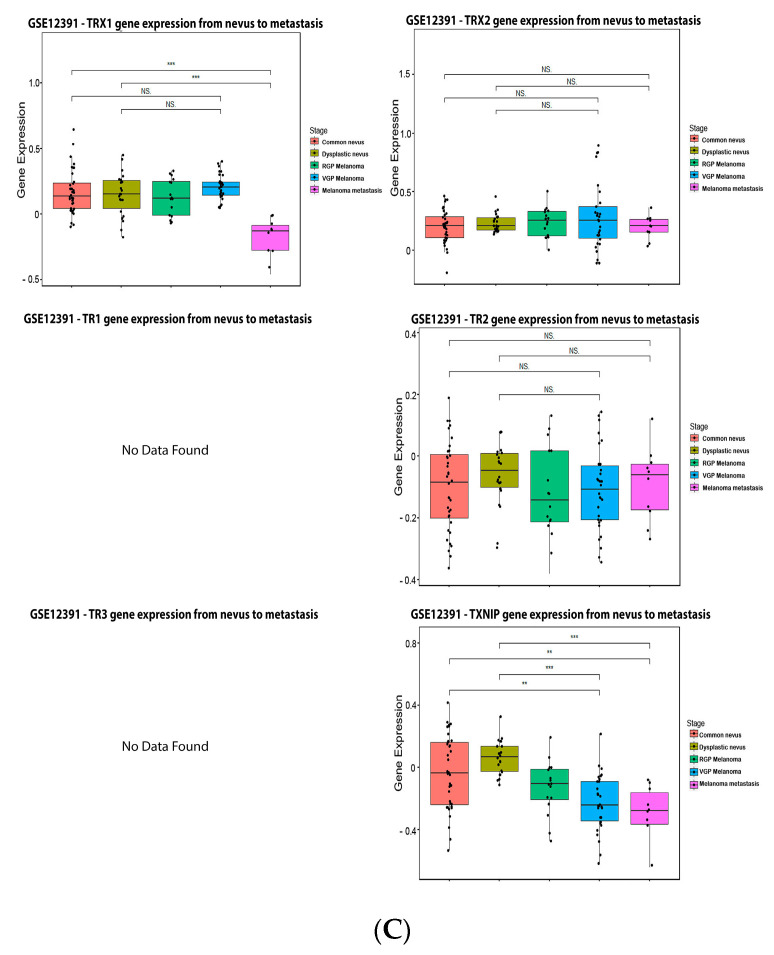
Thioredoxins, thioredoxins reductase and thioredoxin interacting protein on melanoma. (**A**) Percentage of patients with altered genes and types of genetic alterations; (**B**) overall patient survival status, and (**C**) expression in melanoma progression. Red boxes represent the common nevus, yellow boxes the dysplastic nevus, green boxes the radial growth phase (RGP) melanoma, blue boxes the vertical growth phase (VGP) melanoma, and purple boxes the metastatic melanoma. The statistical analysis was performed by ANOVA followed by Tukey test, *** *p* < 0.001, ** *p* < 0.01, * *p* < 0.05 when compared to common or dysplastic nevus. NS: Not significant.

Our data analysis in Figure 6 shows that 34% of the melanoma patients presented alteration on the TRX1 gene, while 7% showed alterations on TRX2, 8% on TR1 and TXNIP, 13% on TR2, and 10% on TR3. The most common alteration was decreased mRNA expression for TRX1, missense mutation for TRX2, increased mRNA expression for TR1-3, and amplification for TXNIP (Figure 6A). Only the altered group of patients with a mutation on TR2 demonstrated significative impact on patient survival (Figure 6B). TRX1 is significantly decreased in metastasis compared to common and dysplastic nevus (Figure 6C), while TRX2 and TR2 showed no differences in expression (Figure 6C). No data for TR1 and TR3 were found about expression in melanoma progression. TXNIP is significantly decreased in VGP and metastasis (Figure 6C).

#### 3.2.5. Peroxiredoxin (PRX)

Peroxiredoxins are thiol dependent peroxidases that catalyze the reduction of hydrogen peroxide, peroxynitrite and organic hydroperoxides [114,115,116,117,118,119,120]. There are six peroxiredoxin isoforms expressed in humans: PRX1, PRX2, and PRX6 in the cytosol and nucleus; PRX3 in the mitochondria; PRX4 in the endoplasmic reticulum; and PRX5 in the cytosol, mitochondria and peroxisomes [119,120,121]. PRXs possess a ‘peroxidatic’ cysteine as an oxidation site, and use the reducing equivalents from the thioredoxin system (TR/TRX/NADPH) or the glutaredoxin systems to regenerate the reduced active form [122]. They are emerging as essential enzymes involved in redox signaling [123,124].

A study with melanoma patient samples showed that PRX1 and PRX2 are decreased in melanoma compared to nevus, and they could be used as biomarkers of the disease progression [125]. Loss of PRX1 may play a role in melanoma progression, which might not be a mere consequence of carcinogenesis [125]. PRX2 is an onco-suppressor in melanoma. Interestingly, PRX2 seems to be downregulated in melanoma, and is able to prevent metastasis. A natural compound with PRX-like activity (gliotoxin) inhibited proliferation, migration and lung metastasis in PRX2-deficient melanoma cells [126]. Epigenetic loss of PRX2 expression was restored by a demethylating agent [127]. On the opposite, enhanced expression of PRX2 inhibited the apoptosis induced by cisplatin or H_2_O_2_ [128]. Recently, PRX5 was proposed as an oncogene in melanoma, conferring to cells resistance against ROS and RNS [129]. PRX6 is overexpressed in most melanomas, and it can promote proliferation by increasing arachidonic acid-dependent lipid signaling [130]. Melanoma cells abundantly express PRX6, and its iPLA2 activity stimulates proliferation. The knockdown of PRX6 resulted in the suppression of cell proliferation and growth. [130]. Alterations in PRXs expression are also associated with antitumor chemotherapy resistance [130,131,132,133,134,135].

**Figure 7:**
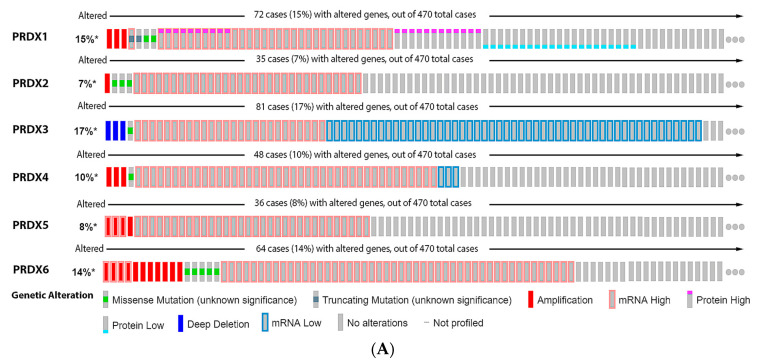

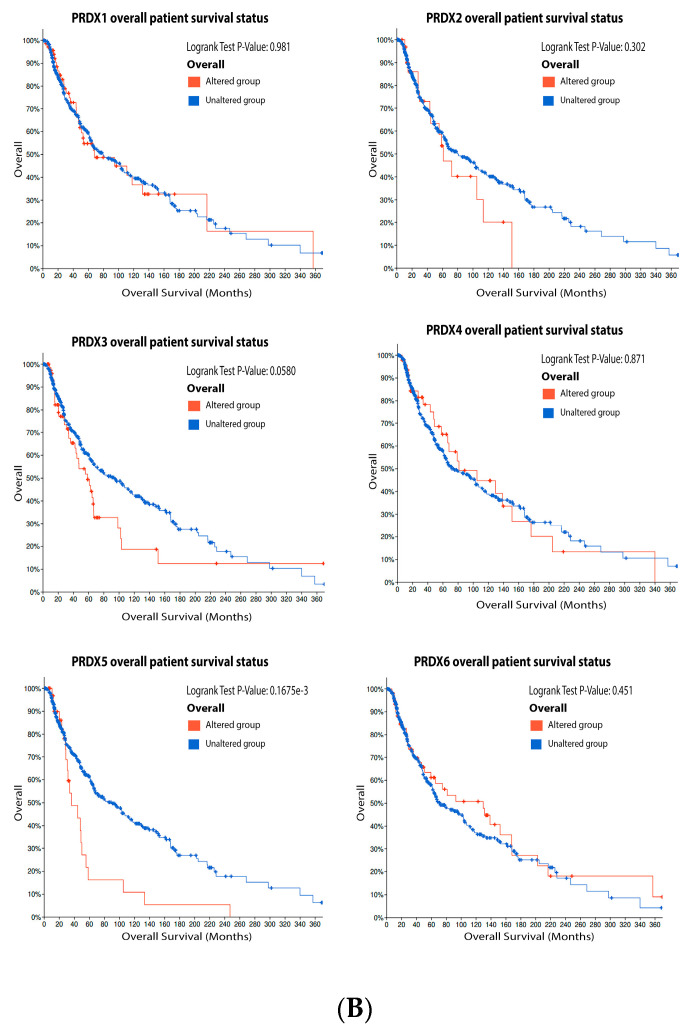

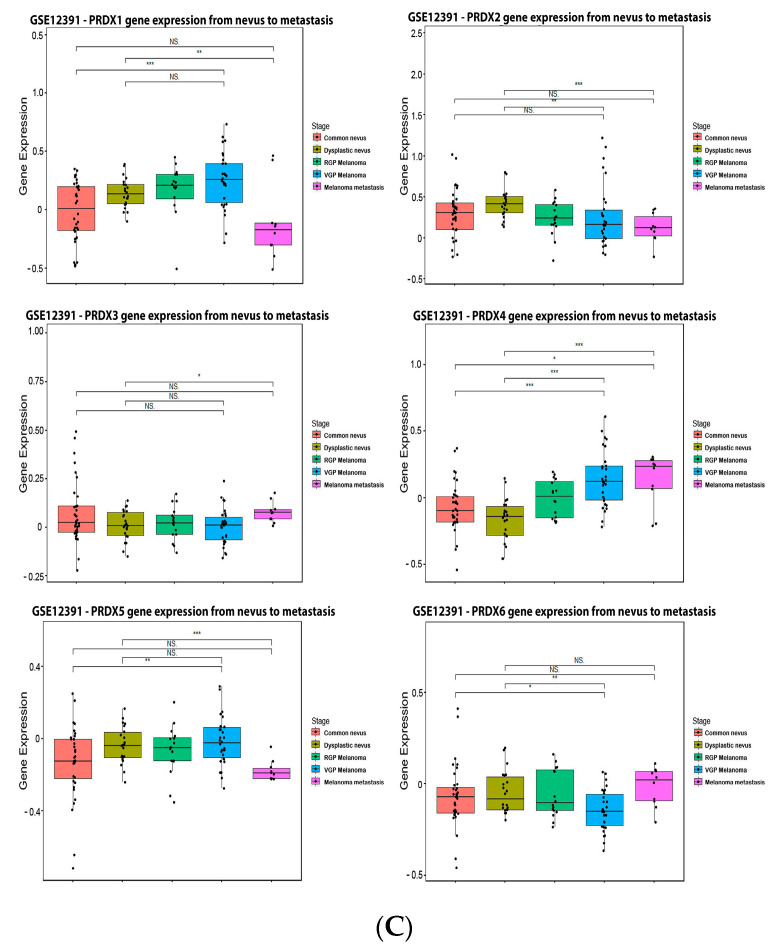
Peroxiredoxins on melanoma. (**A**) Percentage of patients with altered genes and types of genetic alterations; (**B**) overall patient survival status, and (**C**) expression in melanoma progression. Red boxes represent the common nevus, yellow boxes the dysplastic nevus, green boxes the radial growth phase (RGP) melanoma, blue boxes the vertical growth phase (VGP) melanoma, and purple boxes the metastatic melanoma. The statistical analysis was performed by ANOVA followed by Tukey test, *** *p* < 0.001, ** *p* < 0.01, * *p* < 0.05 when compared to common or dysplastic nevus. NS: Not significant.

As observed in Figure 7, 15% of the melanoma patients have alteration on the PRX1 gene, 7% on PRX2, 17% on PRX3, 10% on PRX4, 8% on PRX5 and 14% on PRX6. The most prevalent type of mutation was increased mRNA expression for PRX1, PRX2, PRX4, PRX5 and PRX6, while the most common mutation for PRX3 was decreased mRNA expression (Figure 7A). Alterations on PRX3 and PRX5 had a significant impact on diminishing patient survival (Figure 7B). PRX1 and PRX5 were increased in melanoma vertical growth phase, and decreased in metastatic melanoma, when compared to normal nevus (Figure 7C). PRX2 and PRX6 were significantly decreased in melanoma compared to nevus (Figure 7C), while PRX3 and PRX4 were increased (Figure 7C).

#### 3.2.6. Protein Disulfide Isomerase (PDI)

Protein disulfide isomerase (PDI) is a 55 kDa chaperone, located in the endoplasmic reticulum (ER), whose functions include a thiol oxidoreductase activity, and also the catalysis of formation, breakage and rearrangement of disulfide bonds [136]. Increased PDI expression correlates with cell migration, metastasis, drug resistance [137,138,139], and processes related to ER stress [136]. It has been shown that PDI expression was increased in melanoma patients, when compared to normal melanocytes or non-malignant nevus [56,140,141]. Some anti-cancer treatments target the ER, elevating the content of unfolded or misfolded proteins and ER stress. Unfolded protein response (UPR) signaling can increase PDI expression as well [142,143].

The PDI inhibitor bacitracin increased cell death due to the ER stress induced by fenretinide and velcade [144]. Besides, a melanoma cell line expressing a higher basal level of PDI was less sensitive to the ER stress inducer thapsigargin (THG), and the use of bacitracin increased the sensitivity of these cells to THG [145]. In addition, the incubation of melanoma cells with the cell surface PDI inhibitor rutin led to disorganized cytoskeleton projections and decreased cellular migration. The combinatory therapy with rutin and vemurafenib resulted in a decrease in cell survival when compared to the single treatment [56]. Altogether, these results evidenced the importance of PDI in melanoma, and point to the potential of PDI inhibitors as promising agents for improving the efficacy of chemotherapy in combinatory therapy for melanoma.

**Figure 8:**
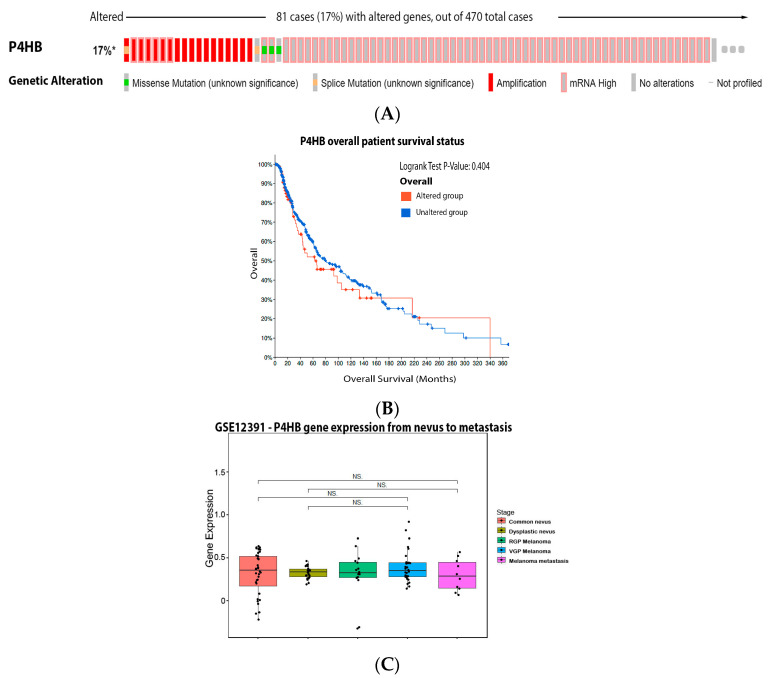
Protein disulfide isomerase on melanoma. (**A**) Percentage of patients with altered genes and types of genetic alterations; (**B**) overall patient survival status, and (**C**) expression in melanoma progression. Red boxes represent the common nevus, yellow boxes the dysplastic nevus, green boxes the radial growth phase (RGP) melanoma, blue boxes the vertical growth phase (VGP) melanoma, and purple boxes the metastatic melanoma. The statistical analysis was performed by ANOVA followed by Tukey test, * *p* < 0.05 when compared to common or dysplastic nevus. NS: Not significant.

Our analysis showed that 17% of the melanoma patients have alteration on the PDI gene, with the most prevalent alteration being increased mRNA expression, but this fact has no impact on patient survival (Figure 8A,B). Despite the literature that showed clear evidence for the role of PDI in melanoma, for our bioinformatics analysis (Figure 8C), no significant alteration was observed in PDI (P4HB) expression among the different stages of the disease.

#### 3.2.7. Peroxidasin (PXDN)

**Figure 9:**
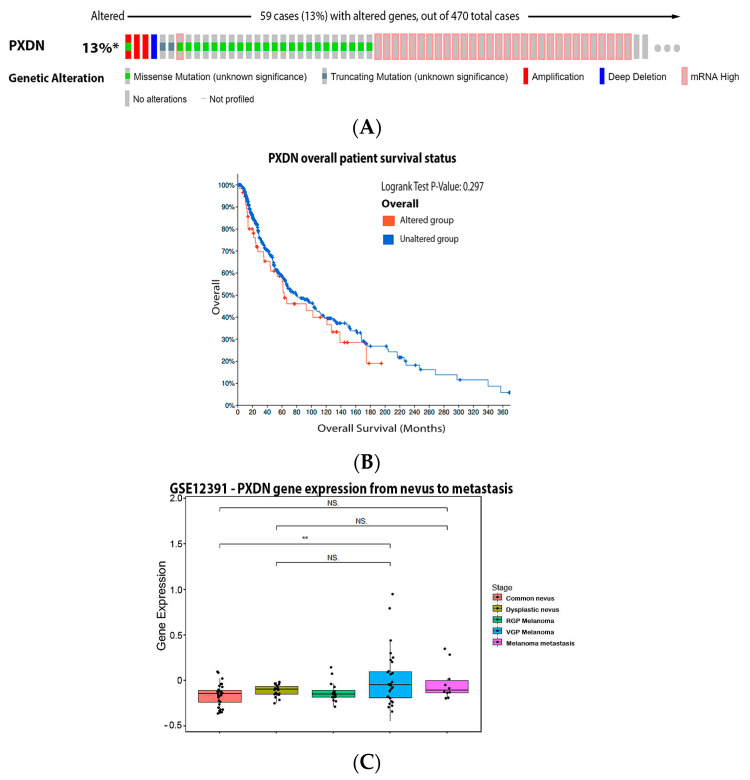
Peroxidasin on melanoma. (**A**) Percentage of patients with altered genes and types of genetic alterations; (**B**) overall patient survival status, and (**C**) expression in the melanoma progression. Red boxes represent the common nevus, yellow boxes the dysplastic nevus, green boxes the radial growth phase (RGP) melanoma, blue boxes the vertical growth phase (VGP) melanoma, and purple boxes the metastatic melanoma. The statistical analysis was performed by ANOVA followed by Tukey test, ** *p* < 0.01 * *p* < 0.05 when compared to common or dysplastic nevus. NS: Not significant.

Peroxidasin (PXDN) was initially named melanoma gene 50 (MG50), due to its expression in melanoma samples, which was characterized as a potent melanoma-associated antigen [146]. PXDN is located in the ER and secreted into the extracellular space. It contains a peroxidase domain and other motifs, typical of extracellular proteins [147]. This protein has been identified as a marker in melanoma, glioma and renal carcinoma, playing a possible role in the regulation of tumor angiogenesis [148]. It is increased in invasive mesenchymal-like melanoma cells, showing high expression in metastatic melanoma tumors. PXDN gene silencing led to reduced melanoma invasion in vitro and inhibited migration in vivo [149]. Our analysis showed that 13% of the patients have alteration on the PXDN gene, with the most prevalent mutation being increased mRNA expression, but it has no impact on patient survival (Figure 9A,B). Corroborating literature data, our analysis demonstrated that PXDN is significantly augmented in VGP melanoma (Figure 9C).

## 4. Discussion

According to our analysis in melanoma patients, all redox proteins have some type of genetic alteration, which evidences their potential role as biomarkers or targets. The most common alteration was increased mRNA expression (78% of the isoforms), followed by a missense mutation (9% of the isoforms) and decreased mRNA expression (9% of the isoforms). Once data about genetic alteration could not be distinguished by the disease or treatment stage, some contradictory data may be found, which reinforces the need for more careful and specific studies. In this sense, more attention should be given to TRX1, once 34% of the melanoma patients have some mutation in this gene. The most prevalent alteration was decreased mRNA expression, which corroborates our data about the expression of TRX1 on melanoma progression that is significatively decreased on metastasis.

Five isoforms showed to be relevant on overall patient survival status: PRX3, PRX5, TR2, GR and NOX4 (15% of the isoforms). The NOX4 gene is altered in 15% of the melanoma patients with the most prevalence for increased mRNA expression and amplification, which corroborates with our data about increased NOX4 expression on melanoma metastasis. Interestingly, however, the group with alteration on the NOX4 gene demonstrated longer survival than the unaltered group. This calls attention to the real role of NOX4 in melanoma. PRX3, PRX5 and GR are antioxidant proteins that contribute to ROS detoxification and not ROS formation like NOX4. The lack of alterations on PRX3, PRX5, TR2 and GR showed to be important for higher patient survival. In fact, PRX5 seems to be decreased in metastasis, while PRX3 and GR seem to be increased at this stage. TR2, however, did not show a significant expression from nevus to metastasis. Again, the lack of more consistent information about the disease stage or if the patient was under treatment or relapse leave some doubts about the real role of these proteins on melanoma. However, it highlights the importance of these redox proteins on survival.

**Figure 10:**
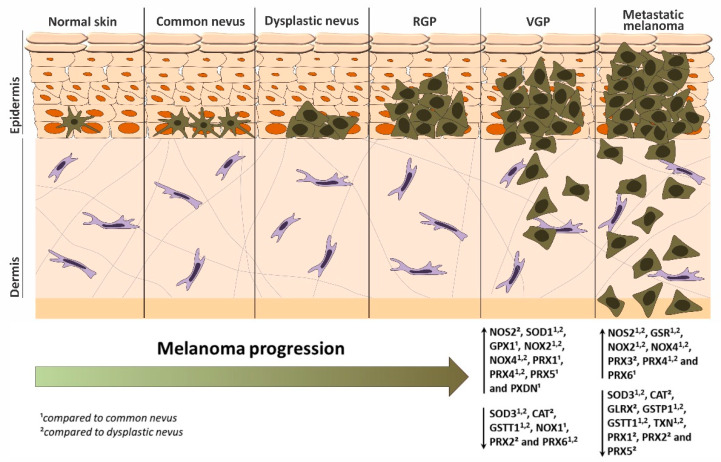
Expression of RNS/ROS-generating and detoxifying proteins in melanoma progression. Thin arrows indicate an increase (↑) or decrease (↓) of proteins in VGP and metastatic melanoma compared to ^1^ common nevus or ^2^ dysplastic nevus. RGP: radial-growth phase. VGP: vertical-growth phase.

We summarized patient data about disease progression obtained from a public microarray database [24], as shown in Figure 10. These results show that the proteins NOS2, SOD1, GPX1, NOX2, NOX4, PRX1, PRX4, PRX5 and PXDN are increased in the vertical growth phase, while SOD3, CAT, GSTT1, NOX1, PRX2 and PRX6 are decreased. Besides, NOS2, GSR, NOX2, NOX4, PRX3, PRX4 and PRX6 are increased in metastasis, while SOD3, CAT, GLRX, GSTP1, GSTT1, TXN, PRX1, PRX2 and PRX5 are decreased. These results evidence that different isoforms of the same protein could play opposite roles in melanoma, such as SOD1 and SOD3; NOX1, NOX2 and NOX4, and all PRXs. Furthermore, the same isoform could be increased or decreased depending on the stage of the disease, such as PRX1, PRX5 and PRX6. These data highlight the importance of discriminating protein isoforms and disease stages in the studies in order to achieve a more precise and complete understanding of the role of each protein, avoiding a non-specific and possible harmful therapy aiming at the inhibition of proteins that are already reduced.

We also summarized literature information found and some analyzed data in Table 1 and Table 2. These data evidence some lack of information about the expression of specific proteins, such as SOD3, PRX4, PRX5, NOX2, NOX3, NOS3, TRX2, TR2, TR3, GLRX1, and GR in melanoma. Additionally, studies investigating the expression of these proteins in sensitive or chemoresistant melanoma are necessary, in which we also observed differences in the expression of some proteins, such as PRX1 and PRX3.

If we consider total proteins and no longer the isoforms, the numbers of alterations become even more evident. We have 100% of the genetically altered proteins, 44% of the proteins impacting on patient survival, and 89% of proteins differentially expressed in disease progression. So, despite the great advance in melanoma field about the redox metabolism in the last years, we still have some gaps in the area that should be investigated further.

## 5. Conclusions

Increasing shreds of evidence point to oxidative stress as one factor responsible for cancer development, progression and metastasis. Treatments with BRAF and MEK inhibitors have been shown to increase ROS in melanoma. Thus, targeting the antioxidant system may contribute to overcoming resistance. Meanwhile, cancer cells are highly adaptive. They cooperate with antioxidant systems to survive in such stressful conditions. By understanding the complex mechanisms involved in adaptation and searching for new targets to make cells more susceptible to treatment, the disease might be overcome. Indeed, exploring the role of redox sensitive proteins and the modulation of redox homeostasis may provide clues to new therapeutic benefits. They may be involved in both disease progression and resistance to chemotherapy. In this sense, they could act as biomarkers, and therefore, possibly as targets to overcome resistance. According to our bioinformatics analysis, all isoforms showed to be genetically altered in some patients, with emphasis on TRX1 which is altered in 34% of patients. The isoforms PRX3, PRX5, TR2, GR and NOX4 demonstrated to impact on patient survival. Besides, iNOS, SOD1, NOX4, PRX3, PXDN and GPX1 are generally increased during melanoma progression, while CAT, GPX3, TXNIP and PRX2 are decreased. These observations are corroborated by the aforementioned literature. For TRX1 and NOX1, our data analysis is paradoxical with some former studies, and this apparent inconsistency might be due to different models used in each case. Our gene expression analysis is derived from biopsies obtained from different patients, while other articles use cell lines or animals. Besides, the stage of the disease could influence the result as well. For example, the levels of PRX1, PRX5 and PRX6 can be increased or decreased depending on the stage. Ultimately, inhibitors or mimetics of these redox sensitive proteins have achieved very promising results. Although no such specific antioxidant therapy is approved for melanoma yet, we foresee that forthcoming investigations on the modulation of these proteins will bring great advances for cancer therapy.

## Figures and Tables

**Table 1:** Expression of redox sensitive proteins in melanoma. NDF: no data found.

Protein	Function	Expression	Inhibitors and Study Model	Effect	References
CAT	Converts H_2_O_2_ to O_2_ and H_2_O.	↓ healthy melanocytes	PEG-Catalase → in vitro, in vivo within mouse xenograft model 3-AT → in vitro	Less proliferation, migration and metastasis, restore intercellular ROS signaling, induce apoptosis.	[67,68,69,71,72,150]
		↑ melanoma patients			
CuZnSOD (SOD1)	Superoxide dismutase-type enzyme; catalyzes the dismutation reaction to convert O_2_^−•^ into H_2_O_2_.	↑ melanoma cell aging	ATN-224 → in vitro; in vivo; and randomized trials in humans DSF/DCC → in vitro	Less proliferation, antiangiogenic activity, and proapoptotic effects.	[63,151,152,153,154,155]
MnSOD (SOD2)		↓ B16F10 cell line; melanoma patients	NDF	NDF	[59]
		↑ melanoma patients			[61,69,151]
ecSOD (SOD3)		NDF	NDF	NDF	NDF
PRX1	Catalyzes the reduction of hydrogen peroxide, peroxynitrite and organic hydroperoxide.	↑ in chemotherapy	Gliotoxin → in vitro, in vivo Frenolicin B → in vitro (colon, breast and lung cancer cell lines), in vivo Conoidin A → in vitro (colorectal cancer cells)	Less proliferation, growth, migration and promotes cell death.	[125,131,132,134,156]
		↓ melanoma compared to nevus			
PRX2		↓ melanoma compared to nevus			[125,126,127]
PRX3		↓ melanoma with methylation			[125,126,127,157]
PRX4		↑ and ↑ in chemotherapy			[133,135]
PRX5		NDF			NDF
PRX6		NDF			NDF
PXDN	Forms a sulfilimine bond through the synthesis of hypohalous acids.	↑ melanoma patients	NDF	NDF	[131]
PDI	Catalyzes the formation, isomerization and removal of disulfide bonds.	↑ melanoma patients	Bacitracin → in vitro Rutin → in vivo, in vitro, in silico	Less migration, cell death and disorganization of the cytoskeleton-including in resistance	[55,140,141,144,158,159,160]
NOX1	Reduces oxygen to superoxide radical (O_2_^−•^) and H_2_O_2_.	↑ melanoma cells	DPI → in vitro Apocynin → in vitro, in vivo VAS2870 → in vitro Honokiol → in vitro, in vivo ML171, GKT136901, GSK2795039, GLX7013114, APX-115 (non-specific for melanoma)	Less proliferation, less cell migration, decreased ROS production.	[19,51,52,54,56,161]
NOX2		NDF			
NOX3		NDF			
NOX4		↑ melanoma cells			
NOX5		↑ melanoma cells			
nNOS (NOS1)	Catalyzes the reaction of L-arginine and oxygen to produce L-citrulline and NO.	↑ melanoma associated with more proliferative profile	L-NAME → in vitro, in vivo L-NIL → in vitro, in vivo L-NoArg → in vitro, in vivo Amg → in vitro, in vivo Smt → in vitro, in vivo Evodiamine → in vitro, in vivo	Less proliferation, growth, antiangiogenic activity, apoptosis.	[33,38,42,44,46,162,163,164]
iNOS (NOS2)		↑ mesenchymal stem cells lacking p53 when exposed to melanoma cells			
eNOS (NOS3)		NDF			
TRX1	Maintenance of a reducing intracellular environment state.	↑ nucleus and cytoplasm	NDF	NDF	[103]
TRX2		NDF			NDF
TR1	Catalyzes the electron transfer to the oxidized form of TRX and other substrates.	↑ melanoma patients	MJ25 → in vitro Auranofin → in vivo NACC → in vitro	Increase ROS levels, induce p53 expression and cell death.	[110,113,165]
TR2		NDF			NDF
TR3		NDF			NDF
TXNIP	Endogenous inhibitor of TRX.	↓ in VGP and metastasis	NDF	NDF	[108,109]
GPX1	Detoxification of organic and inorganic peroxides.	↑ melanoma cells	BSO → in vitro, in vivo, and clinical trials with humans Lomefloxacin → in vitro NBDHEX and MC3181 (derivative) → in vitro, in vivo within xenograft mouse model Ethacrynic acid → in vitro Curcumin → in vitro, in silico	Less proliferation, migration, and induction of apoptosis and overcome melanoma resistance.	[82,83,87,88]
GPX3		↓ melanoma cells			
GST	Catalyzes the conjugation of GSH.	↑ in tumor progression.			[86,90,91,94,166,167,168,169]
		↓ metastasis derived from skin or lymph nodes			
GLRX1	Glutathione-disulfide oxidoreductase activity.	NDF	NDF	NDF	NDF
GR	Catalyzes the reduction of GSSG to GSH.	NDF	2-AAPA → in vitro	Less proliferation, migration, invasion, induction of apoptosis.	[100,169,170]

**Table 2:** Patient data about genetic alteration, survival status and gene expression of redox sensitive proteins in melanoma progression. * compared to common nevus/** compared to dysplastic nevus. NSD: no significant difference. NDF: no data found.

Protein	Genetic Alteration (Out of 470 Cases)	% of Patients with Alteration on the Gene/Main Alteration	Overall Patient Survival (*p*-Value)	Gene Expression	
				VGP Melanoma	Melanoma Metastasis
CAT	45 cases (10%)	10% mRNA high	0.626	↓ **	↓ **
SOD1	68 cases (14%)	14% mRNA high	0.537	↑ */**	NSD
SOD2	34 cases (7%)	7% mRNA high	0.124	NSD	NSD
SOD3	21 cases (4%)	4% mRNA high	0.969	↓ */**	↓ */**
PRX1	72 cases (15%)	15% mRNA high	0.981	↑ *	↓ *
PRX2	35 cases (7%)	7% mRNA high	0.302	↓ *	↓ *
PRX3	81 cases (17%)	17% mRNA low	0.0580	NSD	↑ **
PRX4	48 cases (10%)	10% mRNA high	0.871	↑ */**	↑ */**
PRX5	36 cases (8%)	8% mRNA high	1.675 × 10^−3^	↑ *	↓ *
PRX6	64 cases (14%)	14% mRNA high	0.451	↓ */**	↑ *
PXDN	59 cases (13%)	13% mRNA high	0.297	↑ *	NSD
PDI	81 cases (17%)	17% mRNA high	0.404	NSD	NSD
NOX1	59 cases (13%)	13% mRNA high	0.140	↓ *	NSD
NOX2	34 cases (17%)	7% mRNA high	0.258	↑ */**	↑ */**
NOX3	31 cases (7%)	7% mRNA high	0.576	NSD	NSD
NOX4	69 cases (15%)	15% mRNA high	0.0286	↑ */**	↑ */**
NOX5	33 cases (7%)	7% missense mutation	0.775	NSD	NSD
nNOS (NOS1)	67 cases (14%)	14% missense mutation	0.978	NSD	NSD
iNOS (NOS2)	38 cases (8%)	8% mRNA high	0.618	↑ **	↑ */**
eNOS (NOS3)	83 cases (18%)	18% mRNA high	0.156	NSD	NSD
TRX1	161 cases (34%)	34% mRNA low	0.744	NSD	↓ */**
TRX2	35 cases (7%)	7% missense mutation	0.351	NSD	NSD
TR1	39 cases (8%)	8% mRNA high	0.282	NDF	NDF
TR2	63 cases (13%)	14% mRNA high	7.95 × 10^−7^	NSD	NSD
TR3	48 cases (10%)	10% mRNA high	0.625	NDF	NDF
TXNIP	37 cases (8%)	8% amplification	0.707	↓ */**	↓ */**
GPX1	28 cases (6%)	6% mRNA high	0.846	↑ *	NSD
GPX3	22 cases (5%)	5% mRNA high	0.661	NSD	↓ */**
GSTM1	46 cases (10%)	10% mRNA high	0.526	NSD	NSD
GSTP1	61 cases (13%)	13% mRNA high	0.315	NSD	↓ */**
GSTT1	49 cases (10%)	10% mRNA high	0.806	↓ */**	↓ */**
GLRX1	64 cases (14%)	5% mRNA high	0.404	NSD	↓ **
GR	72 cases (15%)	15% mRNA low	0.0166	NSD	↑ */**

## Data Availability

Data is contained within the article.
